# Biomaterial-Based Additive Manufactured Composite/Scaffolds for Tissue Engineering and Regenerative Medicine: A Comprehensive Review

**DOI:** 10.3390/polym17081090

**Published:** 2025-04-17

**Authors:** Jigar Vyas, Nensi Raytthatha, Puja Vyas, Bhupendra G. Prajapati, Pimpon Uttayarat, Sudarshan Singh, Chuda Chittasupho

**Affiliations:** 1 Krishna School of Pharmacy & Research, Dr. Kiran and Pallavi Global University, Varnama, Vadodara 391240, Gujarat, India; drjigarvyas@gmail.com (J.V.); nensiraytthatha@gmail.com (N.R.); 2 Sigma Institute of Pharmacy, Sigma University, Vadodara 390019, Gujarat, India; pujavyas20@gmail.com; 3 Shree S.K. Patel College of Pharmaceutical Education and Research, Ganpat University, Kherva 3840212, Gujarat, India; bhupen27@gmail.com; 4 Faculty of Pharmacy, Silpakorn University, Nakhon Pathom 73000, Thailand; 5 Centre for Research Impact & Outcome, Chitkara College of Pharmacy, Chitkara University, Rajpura 140401, Punjab, India; 6 Nuclear Technology Research and Development Center, Thailand Institute of Nuclear Technology (Public Organization), Nakhon Nayok 26120, Thailand; pimponu@tint.or.th; 7 Office of Research Administration, Chiang Mai University, Chiang Mai 50200, Thailand; 8 Faculty of Pharmacy, Chiang Mai University, Chiang Mai 50200, Thailand

**Keywords:** additive manufacturing, biomaterials, scaffold, 3D printing, regenerative medicine, tissue engineering

## Abstract

Additive manufacturing (AM), also referred to as three-dimensional printing/printed (3DP), has emerged as a transformative approach in the current design and manufacturing of various biomaterials for the restoration of damaged tissues inside the body. This advancement has greatly aided the development of customized biomedical devices including implants, prosthetics, and orthotics that are specific to the patients. In tissue engineering (TE), AM enables the fabrication of complex structures that promote desirable cellular responses in the regeneration of tissues. Since the choice of biomaterials plays a vital role in scaffold performance as well as cellular responses, meticulous material selection is essential in optimizing the functionality of scaffolds. These scaffolds often possess certain characteristics such as biodegradability, biocompatibility, biomimicry, and porous structure. To this end, polymers such as chitosan, collagen, alginate, hyaluronic acid, polyglycolic acid, polylactic acid, and polycaprolactone have been extensively investigated in the fabrication of tissue-engineered scaffolds. Furthermore, combinations of biomaterials are also utilized to further enhance the scaffolds’ performance and functionality. This review discusses the principle of AM and explores recent advancements in AM technologies in the development of TE and regenerative medicine. In addition, the applications of 3DP, polymer-based scaffolds will be highlighted.

## 1. Introduction

Tissue engineering (TE) is a field that aims to develop functional tissues by combining scaffolds, cells, and bioactive molecules to achieve the desirable tissues’ properties and functions. Originating from reconstructive surgery, the term ‘tissue engineering’ was first introduced by Dr. Yuan-Cheng Fung at the National Science Foundation meeting in 1987 [1]. Since then, there has been a large research development in this field. The first fully tissue-engineered human organ, a trachea, was successfully transplanted into a 30-year-old woman in 2008 to replace the patient’s own damaged airway [2]. An essential aspect of TE involves the utilization of biomaterials as a vehicle to deliver cells and bioactive molecules or therapeutic agents at the target site while providing the structural support needed in the process of tissue regeneration. The choice of biomaterials can be obtained from natural sources or artificially manufactured to mimic the structure of the native tissues. Irrespective of their sources, biomaterials must possess biocompatibility to prevent the activation of immune responses, sterility to guarantee secure incorporation into host tissues, biodegradability to decompose after completing their intended purposes, and bioactivity to stimulate the desired tissue reactions [3]. Regarding biodegradability, the degradation has to be carefully designed such that the materials will undergo degradation at a pace corresponding to the progress of new tissue formation to ensure successful tissue restoration [4]. Besides the aforementioned properties, the surface topography of biomaterials can also influence cellular processes, for example, cell proliferation, cell differentiation, and cell migration [5]. Consequently, the constructed scaffolds with abilities to produce cells with various lineages and hybrid organ architectures are the next challenges in TE [6]. The objective of the review is to discuss the principle of AM and explore recent advancements in AM technologies that utilize polymer-based biomaterials in the development of TE and regenerative medicine. Therefore, this comprehensive review of AM and TE offers a unique perspective on the latest advancements in biomaterial-based AM scaffolds. It highlights the critical role of biomaterial selection in enhancing scaffold functionality and cellular responses, often overlooked in prior literature. Moreover, the review integrates various AM techniques, biomaterial combinations, and their specific applications in TE and regenerative medicine, offering a broader perspective on scaffold optimization. Additionally, the review serves as a valuable resource for researchers and clinicians developing next-generation biomaterial-based scaffolds for patient-specific applications.

## 2. Conventional Scaffold Fabrication

Scaffold fabrication techniques are essential for developing porous biomaterials that support TE applications. Conventional scaffold fabrication methods rely on solvents, heat, pressure, and pore-creating agents to generate interconnected porous structures suitable for cell attachment and proliferation. Commonly employed techniques include solvent casting, freeze drying, thermal-induced phase separation, gas foaming, the sol–gel technique, and electrospinning. These methods have been widely explored for TE, bone tissue repair, drug delivery, and bio-composite scaffold development. Each technique offers unique properties that influence scaffold architecture, porosity, and mechanical strength, making them suitable for specific biomedical applications.

Solvent casting is a cost-effective and user-friendly technique involving the dissolution of polymers such as collagen and chitosan in an organic solvent, followed by the incorporation of an insoluble salt (e.g., hydroxyapatite (HA) or poly(α-hydroxy esters)). The solvent is evaporated, leaving behind a salt–polymer composite, which is then leached with water to create a highly porous scaffold (50–90% porosity). This technique has been effectively used for cardiac TE, bone tissue repair, and bio-composite scaffold fabrication [7]. However, drawbacks include limited mechanical properties, potential residual solvents, and the production of thin scaffolds.

In freeze drying, a polymer is dissolved in a solvent and poured into a mold, after which the solvent is frozen at temperatures ranging from −20 °C to −80 °C and then sublimated under vacuum. This results in highly porous, interconnected scaffolds. The technique is particularly beneficial for applications requiring biocompatibility and high porosity, such as osteoconductive scaffolds for bone TE. However, uneven pore diameters (15 μm–35 μm), a lengthy procedure, high energy consumption, and hazardous solvents remain the limitations of the freeze-drying technique [8].

Thermally induced phase separation involves decreasing the solubility of a uniform polymer solution, causing it to separate into two phases. The low-polymer phase is removed, and the remaining high-polymer phase solidifies into a fibrous network with controlled porosity. Its ability to work at low temperatures makes this technique suitable for integrating biologically active pharmaceuticals into scaffolds without degradation [8]. However, it requires careful selection of polymer–solvent systems and can be complex to implement.

Gas foaming utilizes inert gasses such as methane, carbon dioxide, hydrogen, and nitrogen to generate internal pressure within biodegradable polymers, resulting in gas bubbles and highly porous scaffolds with pore sizes ranging from 30 μm to 700 μm [9]. This method has been successfully employed for biodegradable scaffold fabrication using poly, starch, and biogas blends, producing highly interconnected macroporous structures (in pore diameters varying between 100 and 500 μm). The resulting scaffolds have demonstrated excellent tissue integration and biocompatibility in joint implant models. Both in vitro and in vivo experiments showed that the joint implant model exhibits excellent tissue integration with biocompatibility [10]. Using gas foaming and freeze drying, a macroporous bone scaffold with 70% interconnected and open porosity was developed. The agarose/chitosan matrix was reinforced with nano-HA, proving non-toxic and exhibiting good adhesion and growth in vitro. The apatite formation confirmed its excellent bioactivity, which is essential for implant implants [11].

The sol–gel method is a polymerization process used for manufacturing glass or ceramic-based scaffolds. This approach has been applied to calcium phosphate scaffolds, yielding microporous structures (~260 μm pore size) that promote osteogenesis and bone regeneration [12].

Electrospinning is a technique that utilizes a high-voltage electric field to generate an ultra-fine fiber from charged polymer solutions. The morphology and diameter of these fibers can be controlled by adjusting key factors such as solution viscosity, molecular weight, charge density, and the strength of the electric field. A broad range of nonwoven fibers with diameters from the micron to the nm scale can be produced by electrospinning [13]. The electrospinning method is versatile, allowing for the development of fibers with specific properties by adjusting parameters like voltage, solution viscosity, and needle size, making it suitable for applications like drug delivery and TE.

The starch fusion method involves fabricating porous ceramics using corn-, rice-, or potato-derived starch granules as pore formers, and the binder is low-cost and environmentally friendly. It involves mixing starch granules, ceramic powder, and distilled water to create a suspension maintained at 60–80 °C. The starch undergoes swelling due to water absorption, resulting in a gel-like material that is thermally treated to burn out the organic phase and sinter the ceramic matrix. This process allows for low-dimensional changes during consolidation and drying, allowing for better control of component dimensions. This method was one of the first to process bioactive glasses in porous form, but the low porosity and poor interconnection made it unsuitable for clinical applications. Other polymer phases have been experimented as pore formers for tissue engineering bioactive glass scaffolds [14].

The organic phase combustion method is a strategy for producing porous scaffolds by mixing ceramic powders with a solid polymeric phase of synthetic or natural origin. The blend is pressed to create a “green body” and thermally treated at high temperatures. Upon heating, the polymeric particles fill the space within the component and decompose, while the inorganic particles sinter, resulting in a porous body with a negative replica of the original sacrificial template. To prevent collapse during polymer removal, binders are typically incorporated into the mixture. Both closed and open-cell ceramic foams can be obtained, depending on the volume fraction and nature of the sacrificial polymer. Pore interconnectivity is generally low due to the difficulty in maintaining a homogeneous distribution of polymer spheres. However, scaffolds produced by this method can exhibit high mechanical strength, even comparable to cortical bone. To achieve a highly porous structure, a large proportion of the polymeric phase in the starting mixture is necessary, which can cause cracks in the ceramic body. Therefore, the process needs careful control to avoid defects in the final component [15].

The sponge replication method, patented by Schwartzwalder and Somers in 1963, is a popular and effective method for creating foam-like ceramic scaffolds for tissue engineering. It involves impregnating an open-cell porous template of synthetic or natural material with a slurry of ceramic powder and a binding agent. The sponge is squeezed to remove excess slurry, allowing the sponge struts to be coated with a thin layer of the slurry. After drying, the coated template is pyrolyzed, while the remaining ceramic coating is sintered at higher temperatures to create a porous ceramic with the same architecture as the sacrificial template. The morphological characteristics of the ceramic foam are directly related to the polymeric template used [16].

The thermal integration method involves the creation of porous 3D scaffolds using glass fibers as a starting material. These fibers, with diameters ranging from tens to hundreds of micrometers, are cut and disposed into a mold with porosity originating from the free space between them. A thermal treatment stabilizes the porous structure by thermally bonding the glass fibers, resulting in glass scaffolds with high pore interconnectivity. The final scaffold structure can be tailored based on fiber size, sintering time, and temperature.

Since 45S5 Bioglass^®^ is difficult to draw into fibers without devitrification, other glass formulations, such as silicate and borate bioactive glasses, have been proposed for fibrous scaffold production. A porous scaffold made of glass fibers with a nominal composition of 11.1–12.0 Na_2_O, 15.0–17.1 K_2_O, 2.8–3.3 MgO, 12.7–15.2 CaO, 2.7–3.8 P_2_O_5_, 1.0–1.4 B_2_O_3_, 0.0–0.6 TiO_2_, and 48.5–52.0 SiO_2_ wt.% is currently available as a graft material for bone defect restoration [17]. A detailed comparison of conventional methods for the preparation of scaffolds is presented in Table 1.

## 3. Additive Manufacturing Techniques in Tissue Engineering

AM, which is widely known as 3D printing (3DP), is an innovative technique that builds objects by adding layers one at a time from digital drawings. Unlike the conventional subtractive techniques that rely on the elimination of materials, 3DP adds materials step by step to produce intricate geometries and internal structures, with minimal materials going to waste. AM technology has revolutionized various industries as it reduces time for the development of products/prototypes, decreases production costs, and enables the ability to customize individual products [40]. In the healthcare sector, surgical equipment, prostheses, medical devices, and implants can be tailor-made to suit individual patients. Living cells can also be incorporated during the 3DP process, enabling the technique to be a versatile tool for producing tissues and organs.

### 3.1. Additive Manufacturing in Tissue Engineering

AM is a revolutionary biomedical and TE technique that enables the creation of complex, customizable scaffold structures with precise architectural control. Techniques vary in material compatibility, printing resolution, and mechanical properties, making them suitable for specific TE applications. Understanding these principles is crucial for selecting the most appropriate technique for specific TE applications.

#### 3.1.1. Laser-Based Methods

Laser-assisted 3D bioprinting (LAB) is a non-contact, nozzle-free technology that employs a bioink-laden ribbon guided by laser pulses. The ribbon, reinforced with a titanium or gold layer, absorbs the laser energy and transfers it to propel bioink droplets and cells onto a receiving substrate. This process develops a high-pressure bubble that drives the droplets from the ribbon to the substrate [41]. LAB offers exceptional precision and resolution in fabricating 3D structures, making it suitable for bioprinting micropatterned peptides, DNA, and cell arrays. Additionally, LAB enables the printing of cells at very high densities, comparable to extrusion-based microprinting techniques [42].

**Figure 1:**
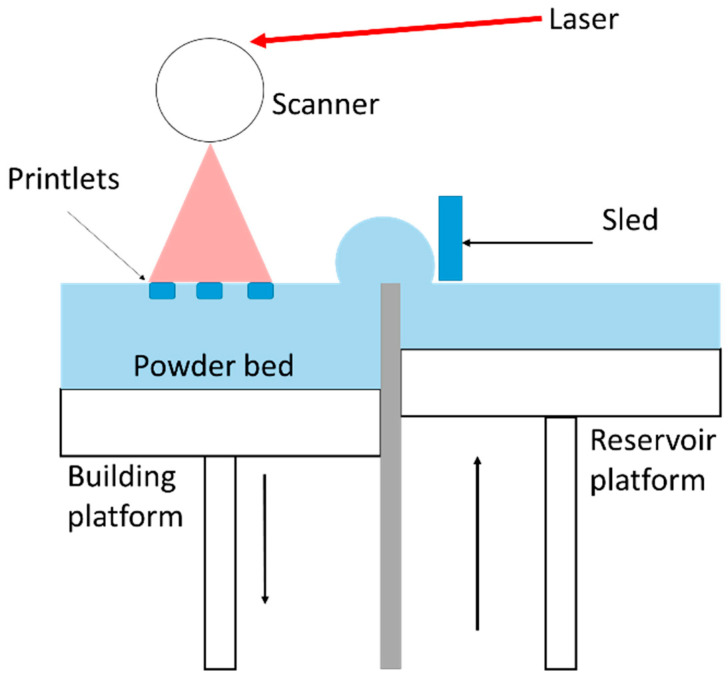
Illustration showing laser-assisted sintering process. The upward arrow indicates the upward movement of the reservoir platform, while the downward arrow indicates the lowering of the building platform. Reproduced with permission from [43] under CCBY.

Selective laser sintering (SLS) is a 3DP technique that employs a high-powered laser to fuse or bind materials into a solid structure (Figure 1). This method uses a CO_2_ laser to fuse powdered material, forming the first layer of the 3DP object. Furthermore, a roller is employed to apply a new layer of powder, increasing the thickness of that layer. Direct SLS production of bio-ceramics is still challenging due to the high-intensity laser induction that requires fast cooling and heating rates. Polymers such as polyamide (PA12 or nylon), thermoplastic polyurethane, and polyetheretherketone are used in scaffold-based composites due to their flexibility, ease of processing, and mechanical properties like elasticity and toughness. They are also easier to process due to their lower melting points and reduced brittleness, making them ideal for SLS applications. Nevertheless, this approach is very advantageous for manufacturing scaffolds-based composite. Elevated temperatures in SLS can cause component breakdown, making it difficult to remove once the scaffold is developed, affecting cell growth and causing inflammatory reactions. However, the resolution of the result depends on the dimensions, configuration, and organization of dust particles, which is the main disadvantage.

**Figure 2:**
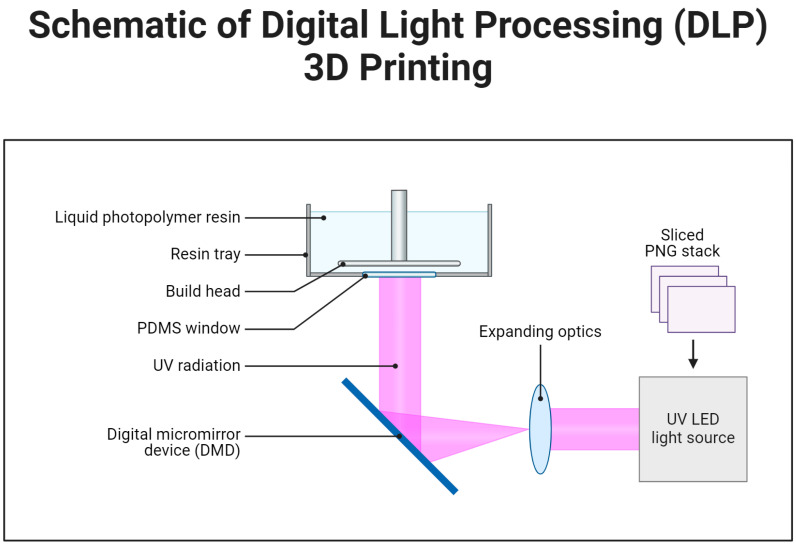
Schematic illustration of digital light processing 3DP. Prepared using BioRender Version 201.

Digital light processing (DLP) is a 3DP technique that uses photopolymer resins to develop a 3D structure by exposing them to a light source. The system includes a digital light projector, a digital micromirror device, a resin tank, and a conveyor system for layer-by-layer construction. The digital micromirror device controls the light beam direction, and each pixel on the display acts as an individual light source, allowing precise illumination of specific resin regions. This process causes the resin to solidify, forming the desired 3D structure layer by layer [44] (Figure 2). DLP offers advantages such as rapid fabrication, high efficiency, and excellent dimensional accuracy, making it suitable for intricate details and complex geometries. Its ability to produce precise and high-resolution structures makes it a valuable tool in TE, particularly for bone, cartilage, and vascular tissues [45].

DLP has limitations due to material constraints and scaffold strength issues. DLP-printed scaffolds may not meet load-bearing requirements and post-processing steps add complexity. DLP is best for small structures, but larger constructs can be challenging due to build volume limitations and extended fabrication times [46].

**Figure 3:**
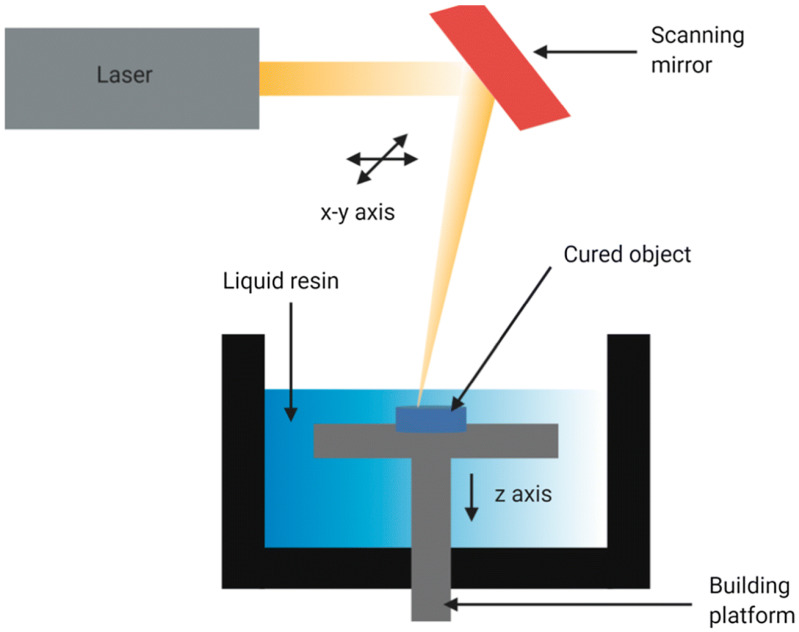
Schematic illustration of stereolithography 3DP. Reproduced with permission from [49] under CCBY 4.0.

Stereolithography (SLA) is a rapid prototyping technique that uses a laser to control the polymerization of bioinks in a layer-by-layer process (Figure 3). The process involves depositing bioink layers and exposing them to UV or visible light, typically through a photosensitive hydrogel using biomaterials such as polyethylene glycol diacrylate (PEGDA) and gelatin methacryloyl [47]. SLA is a powerful tool for developing high-resolution scaffolds by changing parameters such as light intensity, print speed, layer thickness, and exposure time [48]. However, SLA faces limitations in TE due to the poor variety of biocompatible resins available and the difficulty in controlling the resin curing process, leading to potential inconsistencies in scaffold structure and quality.

#### 3.1.2. Non-Laser-Based Methods

Non-laser-based 3DP techniques include methods that do not rely on laser technology to build scaffolds, which can be broadly categorized into extrusion-based and heat-based approaches.

**Figure 4:**
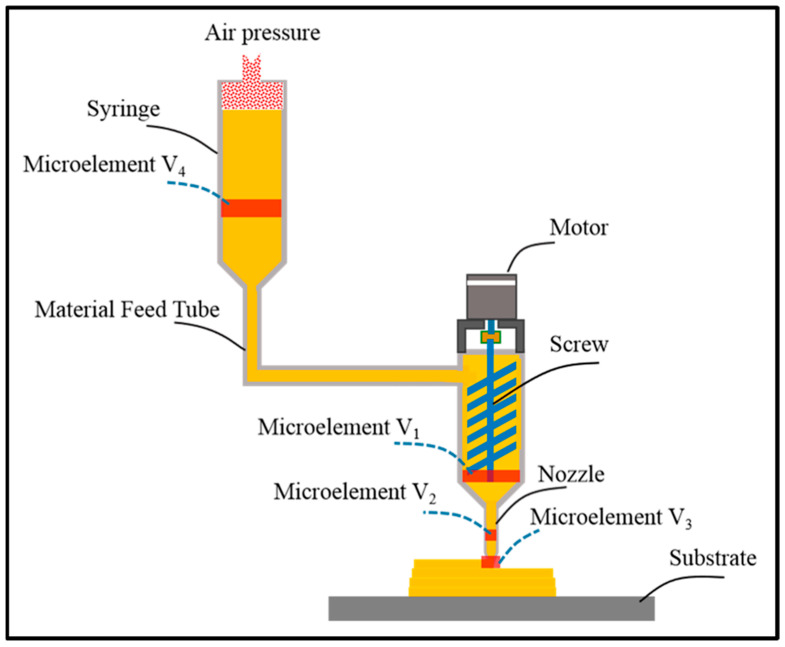
Schematic illustration of extrusion-based 3DP. Microelements V1, V2, V3, and V4 are synthetic biomaterials. Reproduced with permission from [54] under CCBY 4.0.

Extrusion-based technique employs mechanical (piston or screw) or pneumatic forces to release bioink through a nozzle according to a computer-generated pattern (Figure 4). Micro-extrusion printers generate uninterrupted flows of material instead of the individual droplets produced by inkjet bioprinters. The computer-aided design (CAD) software interfaces with the printer to precisely regulate the flow of material streams. Micro-extrusion printers offer a significant advantage in printing high-viscosity bioinks, such as those composed of complex polymers, cell aggregates, and clay-based materials. The key advantages include the precise deposition of highly viscous substances, the ability to create structures with high cell density, and compatibility with a broader range of bioink formulations. This approach significantly broadens the types of bioinks that can be used, making it particularly suitable for densely packed or viscous materials [50]. Micro-extrusion printing is ideal for creating cartilage tissue scaffolds due to its high viscosity, mimicking the dense extracellular matrix (ECM) of cartilage. It is also used for bone tissue scaffolds, providing mechanical strength and biocompatibility with clay-based or polymer-composite bioinks. It is also used in skin graft development, supporting rapid wound healing and regeneration with high cell densities [51]. Vascular TE also benefits from this method, allowing the precise layering of cells and materials to replicate blood vessel structures [52]. However, the major challenge of micro-extrusion printing is that the pressure required to dispense the bioink can lead to the deformation of cellular structures and a reduction in cell viability [53].

**Figure 5:**
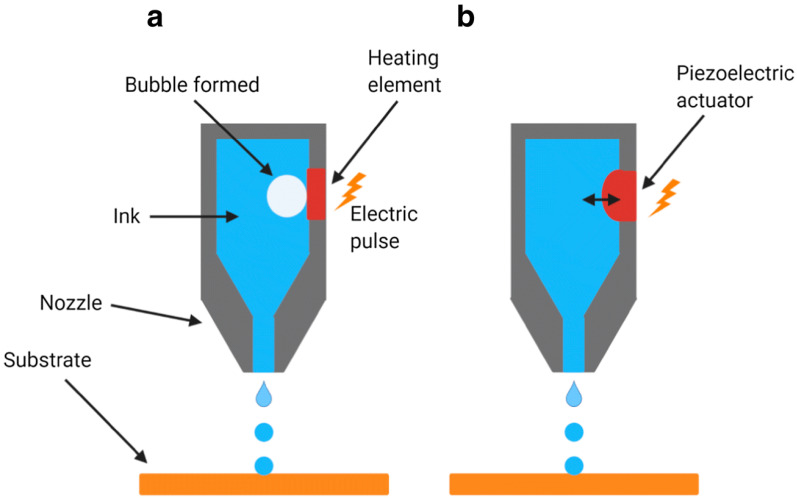
Schematic illustration of thermal (**a**) and piezoelectric (**b**) inkjet printing. Reproduced with permission from [49] under CCBY 4.0.

The 3D inkjet (IJ) printing represents one of the earliest efforts to adapt commercial printing devices for bioprinting, utilizing a modified system to layer biological ink. Inkjet bioprinters, or drop-on-demand printers, use a non-contact method to precisely deposit bioink onto a substrate (Figure 5). Guided by CAD software, these printers accurately recreate tissue structures according to the specified design, making them an essential tool in fabricating printed tissues. The primary advantages of this bioprinting method include its high speed, accessibility, and relatively low cost. However, the technology has certain drawbacks, such as limited droplet size and placement precision, the need for low-viscosity bioinks, frequent issues with nozzle clogging, and potential cellular deformation. In a study by Walczak and Adamski, four different printers were utilized to produce microfluidic components via inkjet printing, achieving a minimum channel size of approximately 200 μm and removing support material in the final step. This approach has been proposed as a viable alternative to traditional rapid prototyping methods [53].

#### 3.1.3. Heat Based Techniques

Heat-based models for 3DP are techniques that utilize thermal energy to shape and deposit materials layer by layer, forming complex structures.

**Figure 6:**
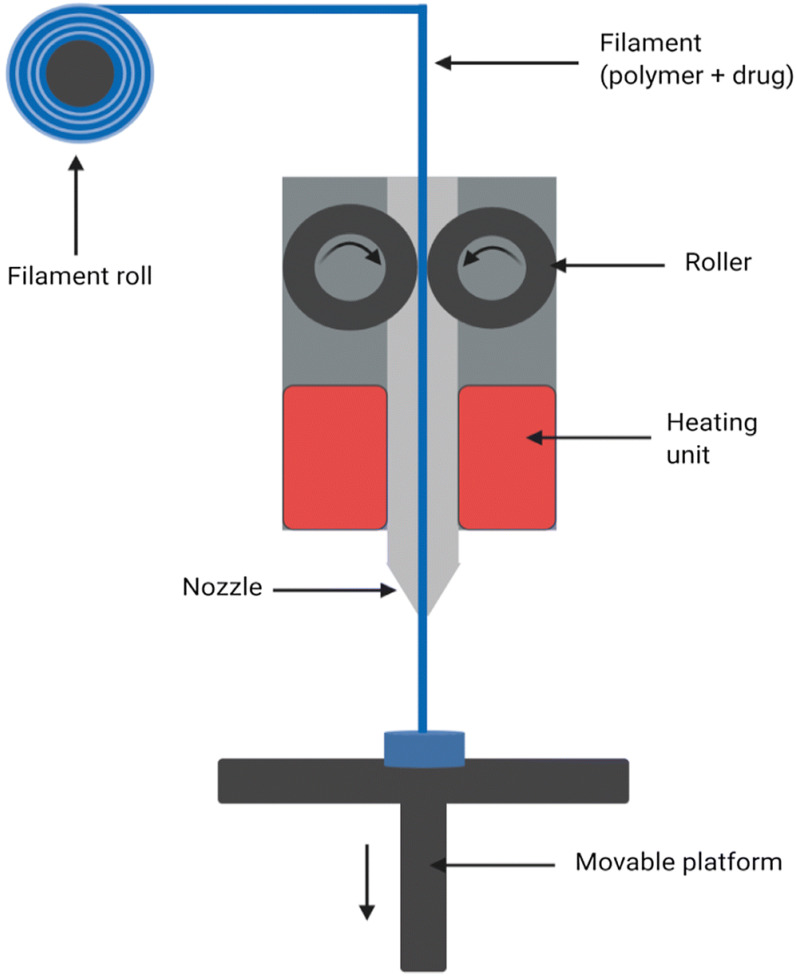
Schematic illustration of fusion deposition modeling-based 3DP. Reproduced with permission from [49] under CCBY 4.0.

Fusion deposition modeling (FDM) is a widely used 3DP method that utilizes thermoplastic polymer filaments to produce 3D objects. The process relies on four key components: the material feeder, print head, gantry system, and build platform [55]. FDM operates through an AM process, where material is deposited onto the build surface layer by layer. In this technique, a thermoplastic filament is fed into an extrusion nozzle that regulates the flow of the material. The filament is heated between rollers, melted, and then extruded through the nozzle onto the build platform to form the object (Figure 6). FDM offers several benefits, including the ability to print with multiple materials, enhanced mechanical properties, improved adhesive strength, and strong mechanical interlocking. However, limitations with the ridged or grooved surfaces cause difficulties in the 3D printing process, which result in moderate accuracy and a relatively slow production process [56].

## 4. Biomaterials Used in Additive Manufacturing

The selection of bioink is crucial in bioprinting, as it directly influences the functionality of tissue constructors. Bioinks consist of biomaterials and cells, with key considerations including printability, mechanical stability, biocompatibility, and biodegradability to support natural tissue regeneration [57]. Biomaterials used as scaffolds fall into three main categories: polymers, ceramics, and metals [58]. Polymers are further classified as natural or synthetic, each offering distinct properties that contribute to the structural integrity and functionality of 3D-printed constructs.

### 4.1. Natural Polymers

Derived from biological substances found in the body, natural polymers are widely used in bioprinting due to their strong bioactivity and ability to interact with cells and microenvironments. They degrade quickly, facilitating natural tissue replacement, and contain motifs that support cell adhesion, proliferation, differentiation, and migration [42,59]. To enhance mechanical strength, these polymers can undergo chemical or physical crosslinking after bioprinting. Commonly used natural polymers include collagen, gelatin, alginate, chitosan, fibrin, and sodium alginate [60].

Collagen, a macromolecular protein abundant in tissues such as cartilage, ligaments, skin, tendons, and bones, consists of polypeptide chains with glycine, proline, and hydroxyproline [61]. Hybrid scaffolds combining 3D-printed collagen and nanocellulose have been developed for tissue engineering, particularly bone regeneration. These scaffolds offer customizable properties like porosity, mechanical strength, and biodegradability, enhancing cell adhesion, proliferation, and differentiation, making them highly promising for regenerative applications [62].

Gelatin, a denatured form of collagen, is a hydrophilic polymer known for its excellent biocompatibility and biodegradability. It supports cell adhesion, migration, and proliferation due to integrin-binding motifs like Arg-Gly-Asp. While widely used in TE applications such as wound healing, cartilage, and bone regeneration, gelatin has limited mechanical strength and thermal stability, often requiring crosslinking agents like glutaraldehyde or genipin [63]. Gelatin methacryloyl [64] hydrogels offer tunable mechanical properties and enhanced printability, making them valuable for bioprinting applications, including vascularized bone and cardiac tissue models [65].

Alginate, a naturally derived anionic polymer from brown algae, is widely used in biomedical applications due to its biocompatibility, biodegradability, and cost-effectiveness [66]. Composed of mannuronic and guluronic acid blocks, its properties are influenced by their arrangement. In 3D printing, alginate readily forms hydrogels through calcium ion crosslinking, enabling the fabrication of scaffolds for bone and cardiac tissue engineering. These scaffolds exhibit favorable mechanical properties, support osteoblast activity, and mimic the mechanical characteristics of native cardiac tissue, highlighting their potential for bone regeneration and cardiac repair [67].

Chitosan, a polymer derived from chitin, is used in biomedical applications due to its biodegradability, mucoadhesive properties, hemostatic properties, and antimicrobial effects [68]. It is used in bone implants and hydrogel wound dressings, with bioactive additives like hydroxyapatite and bioactive glass. 3D-printed chitosan-based scaffolds show promise in skin tissue engineering and wound healing, especially in diabetic models. Chitosan hydrogels with poly(ε-caprolactone) scaffolds improve mechanical strength and osteogenic differentiation [69].

Fibrin is an insoluble protein formed by the polymerization of fibrinogen in the presence of thrombin. Naturally, fibrin possesses bioactive signals that can interact with cells through binding with cell surface receptors. The Arg-Gly-Asp (RGD) sequence in fibrin is crucial for skin cell attachment since it enables cell adhesion and proliferation. Furthermore, in the context of bioprinting, fibrinogen facilitates quick gelation by polymerizing into fibrin, thereby ensuring that the 3D structure of the printed constructs is maintained [70]. Nevertheless, the infrequent utilization of a fibrinogen solution as a standalone biomaterial in bioprinting is mainly attributed to its low viscosity and inadequate shape fidelity [71].

### 4.2. Synthetic Polymers

Synthetic polymers are widely used in bioink formulations due to their tunability, structural consistency, and controlled degradation rates. These engineered biomaterials, such as polycaprolactone (PCL), polylactic acid (PLA), and poly(lactic-co-glycolic acid) (PLGA), offer predictable mechanical properties and support cell attachment and proliferation [72]. Their properties can be modified chemically or blended with natural polymers to create scaffolds that mimic the extracellular matrix while providing enhanced stability, biocompatibility, and porous structures, making them suitable for tissue engineering applications [73].

Polylactic acid (PLA) is a biocompatible and biodegradable polymer used in biomedical applications like sutures, drug delivery systems, and tissue engineering scaffolds. It degrades naturally into non-toxic byproducts, eliminating the need for surgical removal in temporary implants. PLA’s high tensile strength and stiffness make it suitable for load-bearing applications, especially in bone TE and composite scaffolds. Its processability through extrusion, 3D printing, and injection molding enhances its versatility [74]. PLLA has FDA approval for medical applications, and its resins are FDA- and EU-approved for food-related and surgical uses. Sculptra^®^, an FDA-approved dermal filler containing PLA, is used to treat facial lipoatrophy. PLGA, a copolymer of PLA and polyglycolic acid, is widely used in controlled drug delivery due to its biodegradability and biocompatibility [74].

Polyurethanes (PU) are a group of polymers that are made up of oligodiol (soft section) and organic diisocyanate (hard segment) units connected through a urethane linkage. Biodegradable polyurethanes are commonly utilized in biomedical applications because of their excellent mechanical properties and high biocompatibility. The chemical compositions of polyurethanes determine their physiochemical characteristics, including thermo-sensitivity, pH-sensitivity, and biodegradability. Waterborne polyurethanes are commonly produced by adding ionic hydrophilic groups, which convert polyurethanes into an ionomer and enable their dispersion in water. The thermo-sensitivity of polyurethane hydrogels is highly influenced by the compositions of the soft segment oligodiol [75]. Acrylate groups function as a location for ultraviolet curing and can be included in thermosensitive polyurethanes for 3DP.

Polycaprolactone, also known as PCL, is a thermoplastic polymer that has received approval from the FDA for numerous human applications, including bone filling, medication delivery devices, sutures, and TE [76]. This material is a biodegradable polyester that is partially crystalline and breaks down by hydrolysis of its ester bonds under physiological circumstances. PCL is widely considered to be an optimal choice for the construction of tissue-engineered constructs using FDM- technologies. Throughout the printing operations, PCL molecules retain their crystalline forms, which exhibit relatively low to moderate mechanical characteristics. When comparing PCL with PLA and PLA, PCL is more cost-effective, easier to work with, and has greater thermal stability, allowing it to be molded during the melting process.

Polyethylene glycol (PEG) is a synthetic polyether recognized for its hydrophilic, biocompatible, and non-immunogenic properties, making it valuable in tissue engineering [77]. Approved by the FDA for biomedical applications, PEG is commonly used for cell encapsulation. While its hydrogels are inherently non-biodegradable, degradable components can be incorporated to enhance decomposition [78]. Additionally, modifying PEG with cell adhesion domains, such as the RGD peptide or laminin-derived sequences, improves cellular attachment, spreading, and differentiation, thereby enhancing its effectiveness in mimicking the extracellular matrix for regenerative medicine applications [79].

Polylactic-co-glycolic acid (PLGA) is a synthetic copolymer derived from lactic and glycolic acid through ring-opening co-polymerization. It is widely utilized in biomedical applications, including films, porous scaffolds, hydrogels, and microspheres. A common variant, PLGA (75:25), contains 75% lactic acid and 25% glycolic acid, offering superior mechanical strength compared to natural polymers, making it ideal for load-bearing applications. However, due to its low bioactivity, PLGA is often used as a structural support for cell-laden natural polymeric hydrogels, providing an optimal environment for cell growth and tissue engineering applications [80].

### 4.3. Ceramics

Ceramics, though less commonly used than polymers, play a crucial role in replicating rigid tissue structures like bone. Bioactive glasses, calcium phosphates, hydroxyapatites (HAs), and magnesium phosphates are promising bioceramics for bone repair and soft tissue treatment [44]. Bioactive glasses, classified as second and third-generation biomaterials, have gained attention for their ability to convert into HA within the body and form strong bonds with bone and soft tissues. Notable bioactive glass compositions include 45S5 Bioglass^®^, S53P4 BonAlive^®^, and 13-93B3 borate-based glass, which are used in dentistry, bone regeneration, and infection therapy. Their varying chemical compositions demonstrate the tailored design of bioactive glasses for specific therapeutic applications [81].

Calcium phosphate is a bio-ceramic material used in dentistry, bone regeneration, and drug delivery [82]. It is an alternative to the natural bone in orthopedic procedures like titanium implants. Calcium phosphate-based materials are used in periodontal bone regeneration, tooth root repair, and tooth extraction sockets to prevent bone loss. They are also used to repair skull and jaw defects due to their moldability and osteoconductivity [83].

Hydroxyapatite is a naturally occurring inorganic compound composed of 10 calcium ions per molecule, crucial for bone mineralization and structural strength. Its lattice structure consists of six phosphate groups and two hydroxide groups, which can be substituted with fluoride or chloride to modify its properties for specific applications [84,85]. HA is employed to construct scaffolds, used as a coating material for medical implants to improve biocompatibility, used as a coating material for medical implants to improve biocompatibility, and can be integrated with other biomaterials to form customized composite materials. HA is also used to treat bone deformities, cartilage damage, and dental defects. It is now being investigated for its potential use in areas other than bone and cartilage regeneration, including neurons, muscles, and the liver. The regeneration characteristics can be enhanced, and specific cell types can be targeted by modifying them with bioactive compounds.

Tricalcium phosphate is a bioactive material used in bone replacement implants, treating osteoporosis-related fractures, deformities, bone tumors, and osteomyelitis. Its enhanced osteoconductive and osteo-inductive properties promote bone formation and mineralization, aiding regeneration. Tricalcium phosphate forms naturally at physiological temperatures, making it valuable in bone tissue engineering [86]. Bio-cements are biomaterials that mimic the structural and functional properties of natural bone, primarily used for bone repair and regeneration. They are effective bone substitutes due to their ability to set and harden in physiological conditions, forming a solid matrix that supports bone healing. Magnesium phosphate cements (MPCs) are a notable example of these bio-cements due to their superior properties compared to other bone replacements. MPCs form struvite crystals, specifically magnesium ammonium phosphate hexahydrate, which provides excellent mechanical strength and biodegradability [87]. They also show remarkable antimicrobial efficacy, reducing the risk of post-surgical infections. Magnesium, a crucial component of MPCs, is one of the most abundant cations in the human body and plays a vital role in bone metabolism and regeneration [88]. In addition, they exhibited efficacy against many antimicrobial strains. Additionally, magnesium is one of the most common cations in the human body and the most abundant intracellular cation after potassium, naturally found in bone tissues [89]. Due to these advantageous properties, magnesium phosphate cements and their derivatives are considered promising candidates for bone tissue regeneration applications. These properties, combined with their mechanical robustness and biodegradability, make MPCs and their derivatives highly promising candidates for bone tissue regeneration applications [90]. Therefore, is it necessary to differentiate bio-cement and bioglass.

### 4.4. Metals

Metals, in particular titanium alloy, have been primarily used in the 3DP of bone substitutes due to their lightweight nature and exceptional strength [91]. Titanium-based bone implants possess notable characteristics such as significant variations in size and form, intricate structure, and intricate microscopic features. Bone scaffolds processed from titanium alloy have shown excellent biocompatibility, effectively enhancing osteoblasts’ growth and specialization [92]. Bone scaffolds processed from titanium alloy have shown excellent biocompatibility, effectively enhancing osteoblasts’ growth and specialization [93]. Choi et al. [94] utilized CT scans to develop a patient-specific head model and then translated it into a 3DP titanium implant to fill in the damaged skull. This approach significantly reduced the duration of the operation and resulted in no surgical complications during the postoperative follow-up. Although the patient successfully recovered from the infection, further investigation is yet required to ensure the long-term preservation of cell activity and function.

## 5. Design Principles for Scaffold Fabrication

The design of scaffolds involves vital principles such as structural integrity, biochemical cues, control of degradation rates, customization in geometry and porosity, and materials selection. Structural integrity supports cell attachment and growth, while biochemical cues foster cell differentiation and migration. Customization in geometry and porosity is crucial for specific applications and patient needs. These design factors create functional, adaptable, and clinically relevant scaffolds, laying a solid foundation for advanced TE applications [95].

Structural support in TE and regenerative medicine is provided by biomaterials, scaffolds, and the ECM, which work together to support cell attachment, tissue development, and regeneration. Moreover, TE uses scaffolds as structural support to replace damaged or diseased tissues. Typically, the 3D framework mimics the natural ECM with respect to providing a suitable environment for cellular activities. Scaffolds are designed to replicate the composition, architecture, and mechanical properties that serve as a suitable environment for cellular activities. For example, for cartilage tissue engineering, collagen [96], hyaluronic acid [97], and polyvinyl alcohol (PVA) [98] are commonly used materials for cartilage regeneration in TE. Collagen scaffolds, which mimic the natural ECM of cartilage, offer structural support and promote chondrocyte differentiation. Hyaluronic acid enhances tissue hydration and compressive resistance, making it ideal for cartilage regeneration. PVA, a synthetic polymer, improves mechanical strength and elasticity. These scaffolds are crucial for tissue development, especially in large-scale constructs infiltrated with blood vessels for nutrient and oxygen supply. They are designed for controlled degradation, ensuring seamless tissue regeneration, especially in nerve tissue engineering [99]. Cells are another crucial element of bioink in the development of viable tissue constructs. The cell source selection determines the tissue construct’s structure and operational characteristics. The biomaterials and cells work together to influence the architecture and functioning of the bio-printed object [100]. It is important to examine many criteria such as the source of the cells, their intended purpose, the quantity of cells, and their vitality [101]. Stem cells are ideal for tissue regeneration due to their unlimited cell division capacity. They can be enhanced with pericytes to improve bioink stability and functionality. Pericytes stabilize blood vessel formation, promote vascular integrity, and support endothelial cells, facilitating the formation of functional blood vessels in engineered tissue [60]. Bioprinting tissue constructs depend on cellular movement, affecting quality and resolution. Cell migration from bioink via 3DP nozzle affects tissue quality. Single-cell dispersion is ideal for small, intricate tissues, but not for larger, complex ones. Spheroids, 3D aggregates of cells, provide a more robust model for tissue construction, mimicking cell–cell interactions more effectively than single cells [102]. Group delivery of cells or delivering large quantities of cells to a specific target site reduces production time and increases cell viability. HITS-Bio is a high-throughput bioprinting technique that uses a digitally controlled nozzle array to position multiple spheroids simultaneously, enhancing tissue fabrication scalability and cell viability. Cell survival is influenced by the bio-printing technique, factors like crosslinking and porosity, and high printing rates and nozzle pressures. The bioengineer performs structural adjustments, which are reviewed by a physician to verify accurate anatomical characteristics before proceeding to subsequent phases [103]. Tissue-engineered scaffolds are designed to induce cell differentiation within specific tissue types to perform desirable cell functions. They can be modified with biochemical signals, adhesion motifs, and growth factors to facilitate cellular responses. Scaffolds are often constructed using materials that cells naturally interact with, such as collagen, hyaluronic acid, or fibrin. Mechanical properties like stiffness and elasticity are critical in influencing cell differentiation [104]. Scaffold stiffness refers to the resistance of materials to deformation when subjected to an applied force. Cells detect and react to the mechanical characteristics of their surroundings, a process called mechano-transduction. The rigidity of a scaffold can influence stem cell development into lineages, such as osteoblasts and adipogenic differentiation. Customizing scaffold stiffness to align with the mechanical characteristics of the target tissue is essential [105].

Elasticity in 3DP scaffolds is crucial for mimicking native tissue mechanical properties and enabling functionality. Customizing elasticity can be achieved by selecting materials, adjusting designs, and incorporating cross-linking techniques. GelMA (gelatin methacrylate) hydrogels, for cartilage tissue engineering, offer tunable mechanical properties and biocompatibility, allowing researchers to control elasticity to support chondrogenesis and cartilage regeneration [106].

Controlled cell degradation is crucial for successful tissue regeneration, influenced by factors like material composition, structure, processing conditions, and biological environment. Chitosan–gelatin hydrogel is a promising material for cartilage tissue engineering due to its controlled biodegradability, excellent cytocompatibility, and microporous structure formation, making it a potential option for cell growth and tissue repair [107].

In bone TE, 3DP PLGA scaffolds have been used to create structures that gradually degrade over a period of 6 to 12 months, providing customizable mechanical properties and controlled degradation rates, thereby rendering them suitable for critical-sized bone defects and facilitating successful healing and regeneration [108]. Customization of degradation rates can be achieved through material composition cross-linking density (e.g., side groups, aromatic groups, double or triple bonds, and cross-linking) and fabrication techniques (e.g., blending and copolymerization), which controls the degree of chain scission [109].

In terms of customization for patient-specific therapies, TE is revolutionizing healthcare by fabricating functional tissues, with scaffolds being crucial in tailoring therapies to individual patients. These personalized scaffolds are designed to match a patient’s specific anatomical and physiological characteristics, ensuring optimal integration with surrounding tissues. The process begins with imaging, where advanced techniques such as MRI, CT scans, and 3D ultrasound are used to gather detailed patient-specific data. These data are then processed through CAD, creating precise 3D models of the affected tissues or anatomical areas. Finally, the scaffold is fabricated using 3DP, ensuring a perfect fit for the specific location inside the patient’s body. This approach not only promotes effective tissue regeneration but also advances the field of personalized medicine by offering tailored solutions that meet the unique needs of each patient [110].

Biocompatibility and material selection are fundamental design principles in scaffold fabrication. Biocompatibility refers to the ability of a material to fulfill its intended function without inducing toxicity, inflammation, or adverse immune reactions. The selection of biocompatible materials depends on the specific requirements of the tissue being targeted and can include either natural or synthetic options. Among natural polymers, collagen and gelatin [111] are prominent choices due to their excellent biocompatibility and ability to mimic the ECM. Other natural polymers like chitosan [112] and alginate [113] have also been widely used for scaffold fabrication in applications such as skin regeneration, wound healing, cartilage repair, and bone regeneration.

For disease modeling and drug testing, scaffolds in TE are crucial in regenerative medicine, creating models that mimic human tissues and diseases and providing accurate platforms for disease study and drug testing, such as in cancer research using hydrogels or 3DP biomaterials [114]. Structural drug testing provides a more physiologically relevant platform than traditional cell culture systems, allowing researchers to assess drug efficacy, toxicity, and pharmacokinetics more complexly. The 3D tumor models, seeded onto scaffolds, mimic the natural ECM, providing insights into disease mechanisms and testing therapeutic interventions [115]. Researchers have recently developed a 3D neural tissue model using biocompatible materials (alginate, fibrinogen, and gelatin) to mimic the brain’s architecture and support neuronal cell growth [116]. These models are used to study neural stem cell differentiation, improve drug testing accuracy, and evaluate compounds’ efficacy [117].

## 6. Characterization Techniques of Scaffolds

### 6.1. Mechanical Characterization

Due to their mechanical properties, including tensile strength, compressive strength, elasticity, and viscoelasticity, 3DP scaffolds are crucial in tissue engineering. They promote cell growth and differentiation, especially for soft tissues. The mechanical behavior of AM scaffolds depends on factors like material, stress type, geometry, and physical and chemical conditions. Adequate resistance during surgical procedures and maintenance after implantation are essential for load-bearing applications [118]. Tensile strength testing is a crucial method for evaluating the strength, elasticity, and ductility of scaffolds, particularly those designed for tissues like skin or cartilage. It measures elongation and Young’s modulus and provides valuable data for understanding their elasticity and ductility [119].

Soft tissues like skin and cartilage also require scaffolds with viscoelastic properties, which can be assessed using dynamic mechanical analysis. This technique measures how the material behaves under cyclic loading, simulating real-life conditions. The compressive strength plays a significant role in load-bearing tissues such as bone and cartilage. Compressive tests measure a scaffold’s resistance to pressure that presses it together. Briefly, a sample is subjected to increasing compressive force until deformation or failure occurs, yielding data on compressive strength and modulus, suggesting that the scaffold can support physiological loads without collapsing.

Flexural and bending tests are designed to assess how well a scaffold can handle bending forces, providing insights into its stiffness and resistance to bending. In a three-point or four-point bending test, the sample is placed on supports and loaded at one or more points until deformation or failure. The resulting data aid in understanding the flexural strength of the scaffold and modulus. Tests on biomaterials under both dry and wet conditions are essential for assessing their mechanical properties. These tests provide insights into the material’s response in human body settings and its baseline mechanical properties without moisture influence, ensuring its suitability for various biomedical applications [120].

Conducting tensile and compressive tests under both dry and wet conditions is essential for evaluating the mechanical properties of biomaterials, as environmental factors can significantly influence their performance. For instance, studies have shown that mechanical testing under hydrated circumstances at physiological temperatures (37 °C) has been demonstrated to offer insights into how materials respond in settings that closely resemble those of the human body. Additionally, performing tests in dry conditions, such as in a nitrogen atmosphere, helps in understanding the material’s baseline mechanical properties without the influence of moisture. This dual approach allows for a comprehensive assessment of biomaterials, ensuring their suitability for various biomedical applications.

### 6.2. Physico-Chemical Characterization

Beyond mechanical tests, advanced tools like micro-CT imaging and finite element analysis (FEA) provide a closer look at scaffold design and performance. Micro-CT provides high-resolution, 3D images of scaffold structures, enabling detailed analysis of their architecture, porosity, and internal morphology. This non-destructive imaging technique allows researchers to assess the quality and integrity of scaffolds, ensuring they meet the necessary structural requirements for effective bone regeneration [121].

FEA complements other techniques, such as micro-CT, by utilizing computational models to predict how scaffolds may respond to mechanical stresses. By simulating various loading conditions, FEA aids in understanding the mechanical behavior of scaffolds, including stress distribution and potential failure points. This predictive capability is crucial for designing scaffolds that can withstand physiological loads without compromising structural integrity [122]. The combination of micro-CT imaging and FEA provided a comprehensive understanding of the structural and mechanical performance of scaffolds, highlighting the importance of these techniques in scaffold design and optimization [123].

### 6.3. Biological Characterization

Biological characterization of 3DP scaffolds for tissue engineering involves in vitro and in vivo studies. In vitro studies assess cytocompatibility and functionality in cell models, while in vivo evaluations are performed in animal models. In vivo, animal models are crucial for translational and clinical applications, as they create surgical defects for scaffold implantation. Sterilization methods include ethylene oxide gas, ultraviolet irradiation, gamma irradiation, and autoclaving [124]. Protein absorption kinetics involves sterilizing 3DP scaffolds, preparing a protein solution, incubating the scaffolds, and rinsing them to remove unbound protein. Protein quantification is performed using methods like ELISA, BCA, or fluorescence. Adsorption kinetics are studied by repeating the experiment at different time points and plotting the amount of adsorbed protein against time. The experimental data are analyzed by modeling the adsorption process using various isotherms, allowing for insights into adsorption behavior under different conditions. This approach helps elucidate how factors such as protein concentration, scaffold characteristics, and environmental conditions impact adsorption kinetics and efficiency [125]. Cytotoxicity tests evaluate the toxicity of materials to living cells, like 3DP scaffolds. Histological analyses assess tissue regeneration progression, highlighting the scaffold’s role. Histological assessments provide insights into host tissue inflammatory response, biocompatibility, and immune interactions. Biochemical characterization is crucial for assessing tissue maturation and functionality in TE constructs. Cell adhesion rates on 3DP scaffolds are crucial for assessing their effectiveness in tissue formation. Quantitative and qualitative analyses measure speed and security. Cells are seeded onto the scaffold surface and incubated for 1–24 h. Quantitative assessment uses fluorescent dyes for tracking and imaging, while DNA assays like PicoGreen or CyQuant measure adhesion. Cell viability assays assess the metabolic activity of adherent cells [126].

## 7. Applications of Additive Manufactured Scaffolds in Tissue Engineering

**Figure 7:**
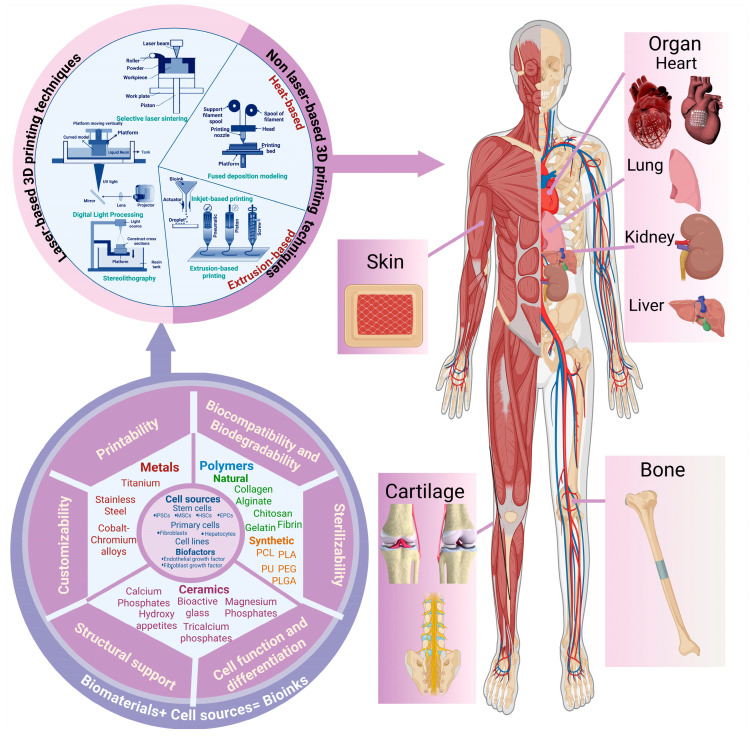
Various 3DP techniques and their applications in TE, emphasizing the utilization of various biomaterials, including metals, polymers, ceramics, and bioinks, to fabricate functional tissues such as heart, skin, cartilage, and bone.

AM has been widely investigated for TE in recent years as a new tool to fabricate scaffolds-based bone TE, skin TE, cartilage regeneration, vascular tissue engineering, and organs [127]. Some of these 3DP scaffolds that are developed into medical devices have shown commercial success in the healthcare system (Figure 7). In the near future, 3DP medical devices that suit individual needs can replace traditional medical products. Specific examples are given below.

### 7.1. Bone Tissue Engineering

**Figure 8:**
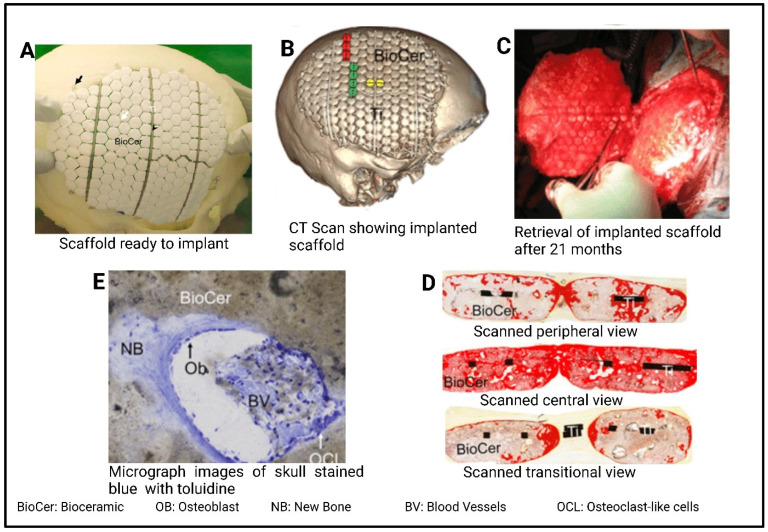
The cranial implant for human use comprises interconnected calcium phosphate tiles supported by a titanium frame, including fixation arms to anchor it to the native skull bone. This design ensures structural stability and bio-integration (**A**). Post-operative CT scans showing the BioCer implant (**B**). The gross image of the retrieved BioCer scaffold after 21 months of implantation, highlighting its complete integration with the surrounding bone tissue (**C**). Microscopic views of the BioCer implant. Top—peripheral view; middle—central view; bottom—transitional view (**D**). Areas with resorption of the BioCer, revealing a typical bone-remodeling pattern, with osteoclast-like cells (OCL) (white arrow) concomitant with an osteoblast (Ob) seam (black arrow), new bone (NB), and BVs (**E**). Reproduced with permission from [132] under CCBY.

Effective bone regeneration and reconstruction are vital to restoring bone structure and function after osteoporosis, fractures, trauma, or neoplastic diseases. This process relies on grafts with critical attributes like biocompatibility, osteoinduction (the process by which materials stimulate the differentiation of progenitor cells into bone-forming osteoblasts), osteoconduction (the ability of a material to provide a scaffold that supports the growth and migration of bone-forming cells), osseointegration (the direct bonding of material to bone tissue without the formation of fibrous tissue), and appropriate mechanical properties [128]. In recent years, bone TE has been significantly advanced, owing to the advent of new technology in the development of new biomaterials together with fabrication techniques that enable the better replication of natural bone tissue with intact biological properties [129]. For example, Ronghuan et al. [130] have investigated the role of controlled porosity in Mg-substituted wollastonite scaffolds, in which Mg substitution not only enhances the bioactivity but also allows for precise tailoring of the mechanical and biological properties of the scaffold. The porosity is controlled during the fabrication process using ceramic stereolithography, enabling the creation of well-defined and interconnected pores. This, in turn, influences bone regeneration by facilitating bone cell growth, proliferation, and differentiation, as well as promoting vascularization within the scaffold. Similarly, Dellavia et al. [131] utilized titanium scaffolds fabricated through selective laser sintering to regenerate alveolar bone in patients with mandibular atrophy and demonstrate a successful regeneration of the titanium scaffold with the surrounding native alveolar bone tissue without notable changes in the bone structure. Omar et al. [132] used 3DP bio-ceramic (BioCer) implants composed of calcium phosphate tiles supported by a titanium frame. The Ti frame in these implants played a critical role by providing structural support and stability, which facilitated better integration with native bone tissue. This design ensured structural stability and bio-integration by enhancing the mechanical performance of the scaffold and enabling effective bonding with the surrounding bone. In the animal studies, BioCer implants and titanium control implants with similar designs were evaluated. The implants were placed in the ovine skulls of sheep and at subcutaneous sites. After implantation, the samples were retrieved at 12 months (skull) and 3 months for detailed analyses using histology, electron microscopy, and Raman spectroscopy. The results demonstrated that BioCer implants promoted bone regeneration even at non-osseous sites. Morphological analyses revealed newly formed bone distant from the host bone, while compositional studies indicated that the BioCer had transformed into carbonated apatite. Histological and ultrastructural analyses confirmed direct bone bonding to the BioCer implants, providing evidence of their osteo-inductive properties. Furthermore, the engineered porous structures of the scaffolds offered microarchitectural cues, which facilitated vascularization and tissue ingrowth into the implant. Proof-of-principle for this approach was supported by a retrieved human specimen, where the BioCer implant had been transformed into well-vascularized osteonal bone. The regenerated bone exhibited a morphology, ultrastructure, and composition indistinguishable from native human skull bone, demonstrating a shift from traditional reconstruction to in situ regeneration (Figure 8).

Additionally, scaffolds infused with osteogenic and angiogenic factors, such as bone morphogenetic proteins and vascular endothelial growth factors, have significantly promoted osteogenesis and vascularization. For example, Kim et al. [133] developed collagen/β-tricalcium phosphate scaffolds loaded with human umbilical vein endothelial cells and adipose stem cells, greatly enhancing both angiogenic and osteogenic outcomes. In another study, Yang et al. [134] fabricated core–shell fibers using poly-3-hydroxybutyrate-co-4-hydroxybutyrate/polyvinyl alcohol and human bone MSC via electrospinning. The bio-electrospinning process preserves cell viability and allows the simultaneous spinning of cells and materials, creating a 3D microenvironment conducive to cell adhesion, proliferation, and differentiation. In vitro assays showed mineralized nodule formation, indicating osteogenic potential. In vivo tests validated the scaffold’s performance, with implants forming bone-like tissue after 16 weeks. This study highlights the potential of coaxial bio-electrospinning technology for creating functional tissue-engineered bone and suggests its future applications. Zhou et al. [135] developed silk fibroin/chitosan/nano-HA (SF/CS/nHA) composite scaffolds combined with autologous concentrated growth factors (CGF) rich in PDGF, TGF-β, and vascular endothelial growth factor (VEGF) to enhance bone defect repair. The 4% SF/CS/nHA scaffold demonstrated optimal properties, supporting BMSC adhesion, proliferation, and osteogenic differentiation, with increased alkaline phosphatase activity, calcium deposition, and upregulated osteogenic markers (Runx2, OCN, OPN) in vitro. In vivo, a rabbit critical bone defect model confirmed that SF/CS/nHA-CGF scaffolds significantly improved bone formation and remodeling, as shown by 3D CT imaging and histological analyses. This combination of materials and CGF offers excellent biocompatibility and regenerative potential for bone TE. These advanced scaffolds demonstrated superior cell adhesion, proliferation, and osteogenic differentiation, successfully promoting bone defect repair in rabbit models.

**Figure 9:**
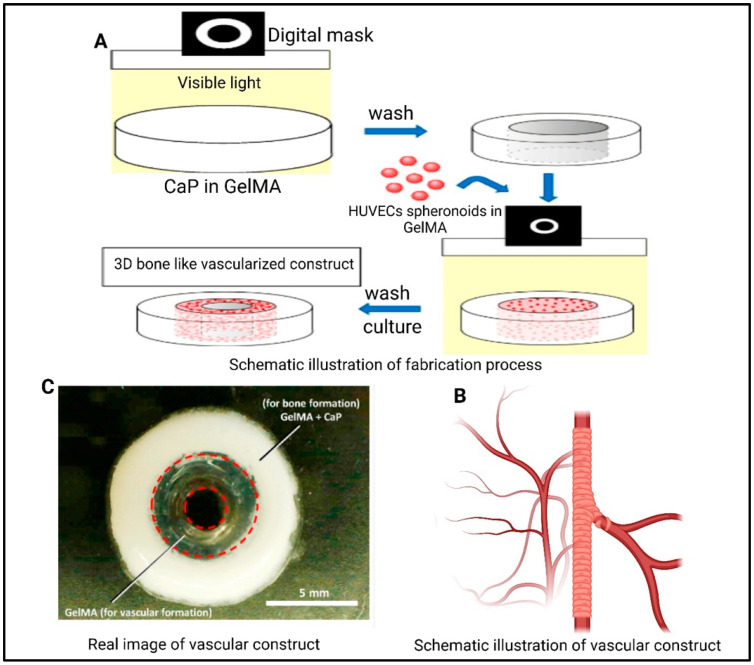
(**A**) Schematic illustration of the fabrication process for 3D hydrogel constructs and a photograph of 3D hydrogel constructs for vascular and bone formation. (**B**) Schematic diagram of vascular construct. (**C**) Real image of vascular construct. Reproduced with permission from [136] under CCBY.

Another study conducted by Anada et al. [136] designed a bone-mimetic 3D hydrogel construct using octa-calcium phosphate (OCP), human umbilical vein endothelial cell (HUVEC) spheroids, and GelMAg hydrogels to replicate the vascularized structure of long bones. The construct featured a dual-ring architecture, with an OCP-infused GelMA outer ring mimicking the dense cortical shell and a GelMA inner ring containing HUVEC spheroids to represent the vascularized bone marrow space. The team employed a two-step DLP fabrication technique to achieve precise spatial patterning and structural fidelity. A refined spheroid culture device facilitated the rapid generation of uniform HUVEC spheroids, which were embedded in GelMA at varying concentrations. The GelMA concentration was found to regulate the development of capillary-like structures from the HUVEC spheroids, demonstrating the material’s tunable properties. Additionally, the inclusion of OCP promoted osteoblastic differentiation of MSC, highlighting its role in bone regeneration (Figure 9). This innovative hydrogel-based construct holds significant promise for advancing bone TE.

### 7.2. Cartilage Regeneration

Cartilage is a flexible connective tissue found in several body areas, including the intervertebral disks, ribs, joints, ear, nose, and trachea. It mitigates the impact of motions and maintains the integrity of the body. The proliferation of cartilage cells and tissue regeneration is restricted due to cartilage being an avascular tissue, resulting in hypoxic situations. Consequently, there is a constraint on the ability of cartilage to regenerate. Cartilage consists of specialized cells known as chondrocytes, which are responsible for the generation of ECM consisting of proteoglycan, collagen fibers, and elastin fibers. Millions of people worldwide suffer from cartilage damage and clinical degeneration that cannot be repaired due to the lack of vascular tissue. Repairing and regenerating damaged cartilage tissue is a crucial concern in the biomedical sector.

Wang et al. [137] developed an injectable hydrogel using gelatin methacrylamide, ε-poly-L-lysine (EPL), and 3-aminophenylboronic acid through a chemical cross-linking technique, with chondrocytes introduced into the hydrogel post-formation. This indicates that the cells were incorporated after the hydrogel was formed, which likely enhanced cell compatibility within the hydrogel matrix. The hydrogel effectively adsorbed proteoglycans secreted by surrounding chondrocytes in situ, forming a supportive microenvironment modified by EPL. Yaqiang et al. [138] developed porous scaffolds with a hierarchical structure, featuring pores of varying sizes at the nano-, micro-, and macro-scales, essential for mimicking the natural ECM of cartilage by enhancing nutrient diffusion, waste removal, and cell integration. The scaffolds were fabricated using bacterial cellulose and decellularized cartilage ECM (DCECM) derived from animal cartilage, where decellularization preserved key biochemical and structural cues for cartilage repair. These scaffolds exhibited exceptional mechanical properties, shape-memory characteristics, and significantly enhanced water absorption, closely mimicking the hydration of native cartilage tissue. Furthermore, the incorporation of DCECM improved the bioactivity of the scaffolds, leading to enhanced adhesion and proliferation of chondrocytes, thereby making them highly effective for cartilage regeneration.

Chondrocytes and MSC are often used with scaffolds to repair cartilage defects. For instance, Li et al. [139] fabricated a nanofibrous poly(ε-caprolactone) scaffold seeded with MSC and treated with TGF-β1, promoting chondrocyte differentiation and enhancing chondrogenesis compared to cell-only cultures. Additionally, this scaffold displayed suitable mechanical properties for cartilage regeneration. In another study, Da Silva et al. [140] seeded MSC onto PCL-nanofiber scaffolds and cultivated them in a multi-chamber flow perfusion bioreactor. The bioreactor significantly enhanced the production of cartilaginous ECM, boosting chondrogenic differentiation. Heirani-Tabasi et al. [141] developed an injectable chitosan–hyaluronic acid gel loaded with adipose-derived MSC and chondrocyte-derived extracellular vesicles. This gel increased the expression of chondrogenic genes and proteins, such as Col II, promoting superior cartilage regeneration in a rabbit osteochondral defect model compared to cell-free hydrogels. Rathan et al. [142] utilized a bioprinting technique to create cartilaginous tissues. They used a bioink composed of alginate functionalized with cartilage ECM, which supported mesenchymal stem cell growth and significantly improved cartilage tissue formation.

### 7.3. Skin Replacement

The technique of 3D bioprinting has emerged as a transformative advancement in human skin TE, enabling the development of functional and naturally layered skin structures. These bio-printed constructs are designed to mimic the architecture of native skin, comprising layers such as the epidermis, dermis, and hypodermis. Unlike conventional methods, 3D bioprinting provides a cost-effective and scalable approach for producing skin substitutes that offer long-lasting functionality and reliability post-implantation, demonstrating their potential for transplantation and regenerative medicine applications [143]. The integration of hydrogels in 3D bioprinting has further enhanced its versatility, particularly in the development of cellular microfluidic channels. These channels are critical for replicating vascular networks and ensuring efficient nutrient and oxygen transport within the bio-printed skin. Hydrogels provide structural support and maintain cellular functionality, making them indispensable in the fabrication of vascularized constructs that mimic the complexity of human skin [144].

In the field of skin grafting, 3D bioprinting offers a promising alternative to traditional donor-dependent methods. By printing blood vessels and skin-like structures, this technique eliminates the need for donor tissues, thereby reducing patient discomfort and improving therapeutic outcomes. Additionally, 3D bioprinting enables the customization of biological scaffolds, tailoring grafts to individual patient needs and enhancing integration with host tissues [145].

Advances in TE have revolutionized wound healing, yet significant challenges persist in replicating full-thickness human skin for severe injuries. Bioprinting technology offers a promising solution by fabricating complex, multi-layered skin structures capable of enhancing wound closure and promoting tissue regeneration. Among these advancements, the ability to restore normal collagen remodeling, a critical component of functional skin repair, remains underexplored. Jorgensen et al. [146] conducted a study to evaluate the effectiveness of bio-printed skin in improving full-thickness wound healing and achieving normal collagen remodeling. By leveraging a bioink composed of human keratinocytes, melanocytes, fibroblasts, dermal microvascular endothelial cells, follicle dermal papilla cells, and adipocytes, they developed a tri-layer skin structure. This innovative construct was tested on full-thickness wounds in athymic mice, providing insights into its potential to accelerate epithelialization and restore human-like skin properties.

For burn injury treatment, tissue-engineered skin is being extensively investigated as a clinical solution to improve wound closure and patient quality of life. Three-dimensional bioprinting has demonstrated success in healing deeper burns, offering a reliable and effective method for reconstructing damaged skin. The ability to produce complex, patient-specific skin structures ensures better functional and esthetic outcomes in burn patients [147]. Moreover, 3D bioprinting is revolutionizing the treatment of severe burns by providing highly customized and complex skin substitutes. Through advanced scanning technologies, bioprinters can fabricate skin that is anatomically and biologically tailored to each patient. This approach not only enhances functional outcomes but also enables the formation of tissues and organs using living cells from bioinks, bridging the gap between regenerative medicine and personalized therapy [148].

### 7.4. Vascular Tissue Engineering

Vascular disorders, particularly cardiovascular diseases, are a major global mortality cause [149]. Autologous harvesting, such as saphenous veins, is used to replace small blood vessels, but site morbidity remains an issue [80]. Therefore, the fabrication of vascular-like materials to resemble the structure and characteristics of blood vessels has emerged as the optimal solution. The creation of tissue-engineered blood vessels involves developing scaffolds with mechanical properties that support the elasticity and ability to withstand burst strength as the natural blood vessels.

A porous tubular scaffold is developed, and specific cells are cultivated. The scaffold rapidly degrades, leading to the release of ECM proteins. Following this, the matured structure is conditioned in a bioreactor and stored in a buffer solution until ready for in vivo implantation [150]. Networks are formed through the aggregated formation of multicellular vascular tissue spheroids, which can start with a high density of cells and mimic the target tissue’s functional and architectural characteristics. Spheroids can be fabricated using various techniques, including micro-molding, cell sheets, microfluidics, rotating wall vessel techniques, pellet culture, hanging drop, spinner culture, liquid overlay, and external force. They can fuse and assemble into macro-tissues through cell-to-cell adhesion. A study by Norotte et al. explored an innovative method to develop vascular grafts using multicellular spheroids and a rapid prototyping 3D bioprinting approach. This technique eliminates the need for traditional scaffolds, which can sometimes cause unwanted immune reactions or hinder the natural interactions between cells. Instead, the study used vascular cells like smooth muscle cells and fibroblasts to form small, spherical units called spheroids or cylindrical cell aggregates. These cellular building blocks, measuring between 300 and 500 μm in diameter, acted as the raw materials for the vascular constructs. The process involved bioprinting these spheroids layer by layer around temporary molds made from agarose rods, which helped shape the structure during the initial stages. Over time, the printed cell units fused together naturally, forming continuous vascular tubes. These tubes could be customized to have one or two layers and varied in diameter from 0.9 to 2.5 mm. The technique stood out for its ability to create vascular structures with complex branching patterns and varying diameters, offering a scalable and versatile approach for engineering blood vessels without relying on traditional scaffolds [151].

### 7.5. Organs

By enabling precise control over cell placement and material composition, 3DP technology has had a significant impact on biomedical engineering, enabling researchers to mimic human tissues’ complex structures and functions [152]. Notable advancements in 3DP have been made across various organs including the heart [153], kidney , liver [154], and lungs [155].

Heart: The transplantation of tissue-engineered heart devices has emerged as a promising solution for patients suffering from advanced congestive heart failure and severe coronary artery disease. In an innovative study, Mehrotra et al. [156] developed a bioink made from non-mulberry silk that incorporated CNT and HUVECs. This bioink was carefully designed to improve both the electrical conductivity and structural integration of a bio-printed heart device. The heart device aimed to replicate the anisotropic characteristics of native cardiac tissue, which are vital for the proper conduction of electrical signals and mechanical contraction in the heart [157]. An anisotropic heart device refers to a structure that mimics the natural arrangement of cardiac muscle fibers. The bio-printed heart device also encourages vascularization, essentially the formation of new blood vessels by integrating HUVECs, which is critical for angiogenesis. Additionally, the device is engineered with GelMA microspheres that are infused with calcium peroxide and IL-10, substances aimed at addressing damaged cardiac tissue. Calcium peroxide plays an important role by releasing oxygen, which is crucial for tissues suffering from ischemia, while IL-10 helps modulate the immune response and fosters tissue repair. These microspheres are designed to enhance the regenerative capacity of the device, enabling it to replace and repair damaged heart tissue [158].

**Figure 10:**
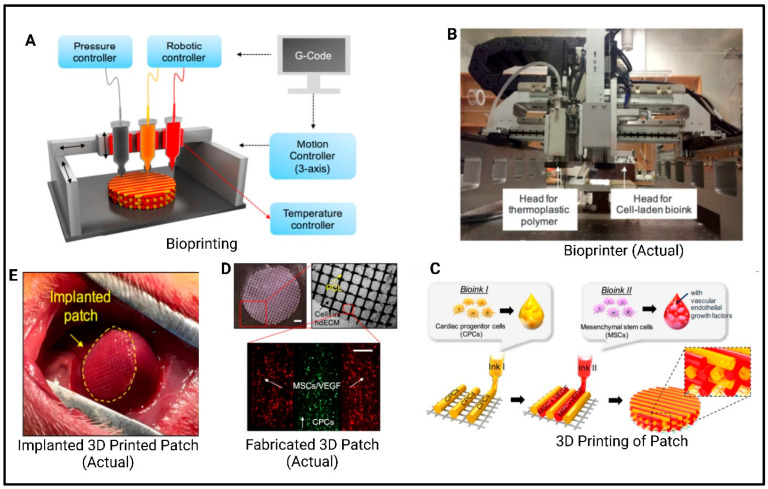
Schematic of pre-vascularized stem cell patch. (**A**) Illustration of 3D cell printing system, and (**B**) macroscopic view of the printer. (**C**) Illustration of pre-vascularized stem cell patch including multiple cell-laden bioinks and supporting PCL polymer. (**D**) Fabricated patch including the two types of cell-laden bioink and PCL supporting layer. (**E**) Optical image of the implanted patch. Reproduced with permission from [160] under CCBY.

One of the key components contributing to the success of this tissue-engineered heart device is the use of carbon nanotubes (CNT). These nanotubes significantly enhance the mechanical properties of the bio-printed heart structure, particularly by mimicking the electrical and mechanical environment of native cardiac tissue. Their high electrical conductivity helps ensure synchronized electrical signal propagation, while also enabling the device to withstand the stresses and cyclic loading typical in cardiac environments. Moreover, the CNT interacts with cardiac cells, promoting their alignment and differentiation—two factors that are critical for developing functional cardiac tissue [159]. The study by Liu et al. [160] used three biological inks to create a myocardial mesh with multiple structures. The patch was supported by a PCL scaffold and printed with two bioinks: one containing heart-derived cytoplasmic matrix bioink containing cardiac group cells (CPC) and the other containing MSC and VEGF. The printed structure improved cell interaction and differentiation promoted vascularization through the WT1-mediated epicardial Wnt/β-catenin signal, reduced cardiac fibrosis, and promoted cardiomyogenesis and neovascularization in damaged myocardium, as shown in Figure 10.

**Figure 11:**
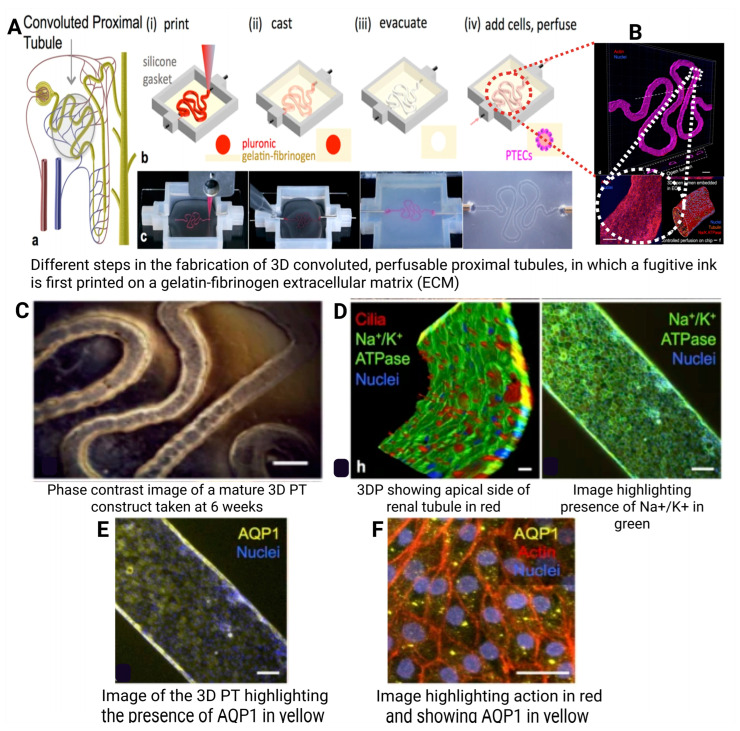
(**A**) Manufacturing strategy using sacrificial 3DP to create hollow channel after post-encapsulation with a gelatin–fibrin ECM hydrogel. (**B**) Proximal tubule-like cell fully epithelialized after post-seeding and culturing with proximal tubule epithelial cells. (**C**) A phase contrast image of a mature 3DP construct taken at 6 weeks. (**D**) 3D rendering of a partial tubule showing the apical side, which highlights the primary cilia (red), and an image of the PT highlighting the presence of Na/K ATPase in green. (**E**) Image of the 3DP, highlighting the presence of (Aquaporin 1) AQP1 in yellow. (**F**) Image highlighting actin in red and showing AQP1 in yellow. Reproduced with permission from [162] under CCBY.

Kidneys: Bioinks containing renal cells have enabled the creation of kidney tissue models for drug testing and toxicity studies. Research is progressing towards producing viable nephron structures. Studies have highlighted the significance of 3DP in treating renal calculi or kidney stones. Xu et al. [161] fabricated 12 patients to simulate percutaneous nephrolithotomy, by selecting three puncture points from the kidney’s upper, middle, and lower calyx. They recorded the gypsum and silicone layer-by-layer-printed stone clearance rate and used the puncture site with the highest clearance rate in the surgery. The results showed a high correlation between postoperative calculus volume and the models, demonstrating the potential of 3DP and in vitro simulation technology in selecting calyces and establishing the best renal puncture channel for complex calculi treatment. In this study, 3DP played a crucial role in preoperative planning, particularly in simulating surgical approaches and evaluating different puncture sites. The 3DP kidney models enabled a detailed analysis of the kidney’s anatomy, allowing for the selection of the most optimal calyx for puncture, which is vital for successful stone clearance. The ability to simulate the procedure prior to surgery demonstrated the potential of 3DP models for improving surgical outcomes, especially for challenging cases such as full-staghorn stones. As shown in Figure 11, a bioprinting method has been developed to create 3D human renal proximal tubules in vitro, embedded within an ECM comprising a network of gelatin and fibrin and housed in per-fusible tissue chips. These tubules, characterized by proximal tubule epithelial cells, can be maintained for over two months. The 3D proximal tubules show improved epithelial morphology and functional properties compared to 2D controls [162].

Liver: The technique of 3DP has significantly advanced liver transplantation by enabling the development of patient-specific liver models that replicate complex anatomical structures, including vasculature, thereby enhancing preoperative planning and surgical precision. The fabrication process involved converting enhanced CT imaging data into 3D models using CAD software. These models were then printed using advanced 3DP, such as SLA printers, which offer the high resolution and precision necessary for replicating intricate liver anatomy. Materials like photopolymerizable resins are employed due to their ability to accurately mimic the mechanical properties of liver tissue [163]. In a pioneering study, researchers developed a protocol for 3DP synthetic livers, replicating the native livers of patients undergoing living-donor liver transplantation. These models accurately represented the complex networks of vascular and biliary structures, facilitating better preoperative surgical planning and potentially improving patient outcomes [164].

**Figure 12:**
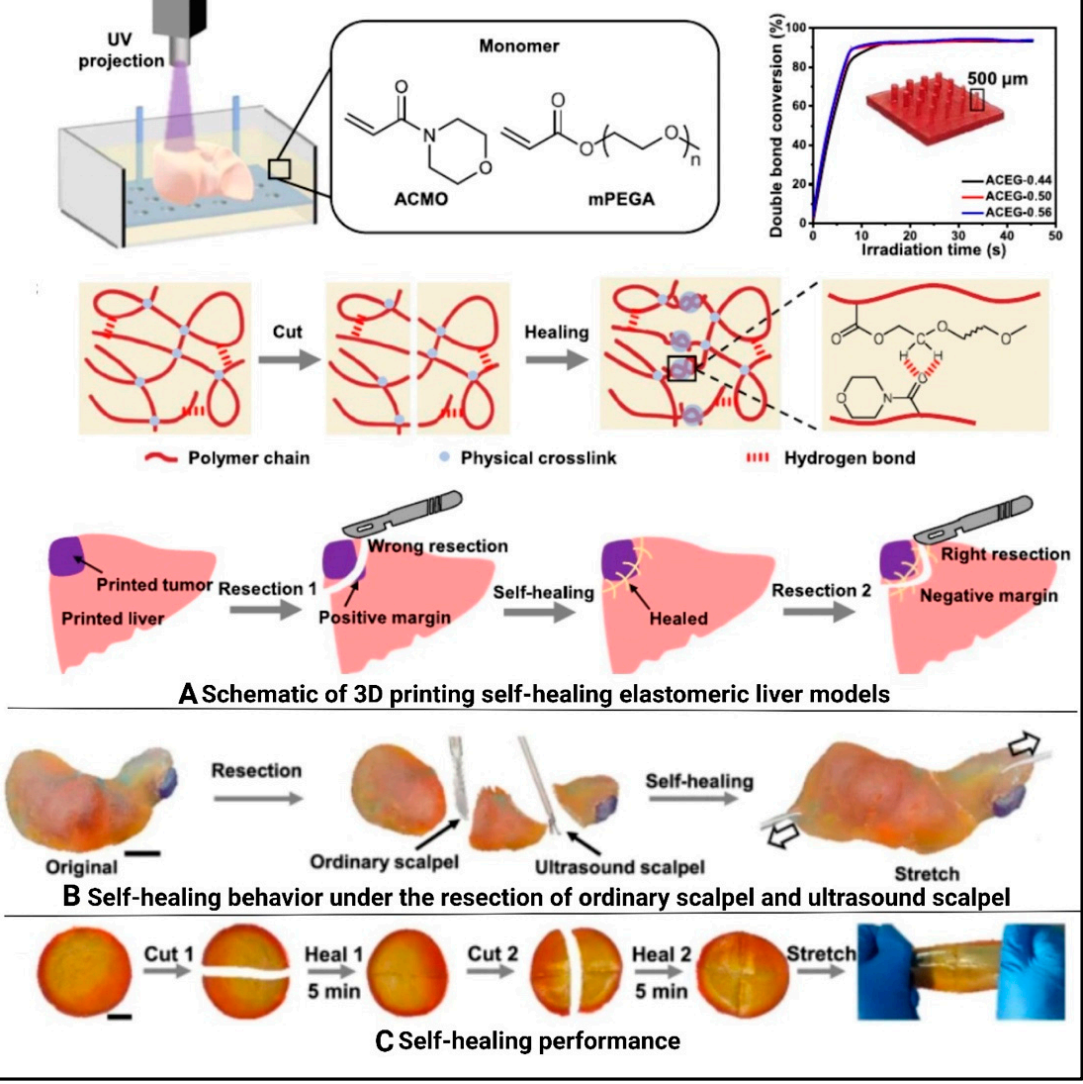
Diagram illustrating 3DP method for self-healing elastomeric liver models (**A**) and self-healing behavior (**B**). Demonstration of a healed sample being deformed (**C**). Reproduced with permission from [163] under CCBY.

In a notable clinical study conducted at the Department of Surgery Nagasaki University, Nagasaki, Japan, a 3DP liver model was used to facilitate preoperative visualization of anatomical structures, including the venous network, enabling tailored surgical plans that improved the safety and efficacy of the transplantation procedure. The 3DP liver model was fabricated by SLA using photopolymerizable resins, with CT imaging data providing detailed insights into the liver’s volume, graft size, and vascular architecture. This 3DP liver model was developed to optimize liver transplant surgery. The model predicted graft size, volume, and venous drainage, reducing operative time and blood loss. The model visualized anatomical structures in 3D, reducing operative time and complications [165]. SLA-based 3DP of customized liver models using crosslinked self-healing elastomers offers a short printing time through CT examination. These models support repetitive resection for optimal trace through a trial-and-error approach. The preliminary explorative clinical trial (NCT06006338) includes five participants for preoperative planning, demonstrating promising application value in clinical practice [163], as shown in Figure 12.

Lungs: The primary function of the lungs is gas exchange, with alveoli being crucial for this process. Three-dimensional bioprinting of alveoli-mimicking structures is challenging due to alveoli’s complexity, size scale (~200 μm), and air–blood barrier (0.62 μm). Two pioneer studies investigating 3DP alveoli-like structures have been conducted. Horvath et al. [166] printed a gelatinous protein mixture (Matrigel™) with endothelial and epithelial cells mixing with the protein precursor to be printed out in the final scaffold. Such a labor-intensive approach enables the thinner and more homogeneous cell layers to be achieved by conventional techniques, wherein cells are typically seeded in a labor-intensive process. In the conventional manual method, cells are deposited individually or in small groups, often leading to uneven distribution and variable layer thickness. Although the barrier (here “barrier” refers to the air–blood tissue barrier (also called the alveolar-capillary barrier), which is a critical interface in the lungs) was unsuitable for implantable engineered tissue translation, it provided the potential for in vitro toxicology and pharmaceutical assessments for basic lung studies.

**Figure 13:**
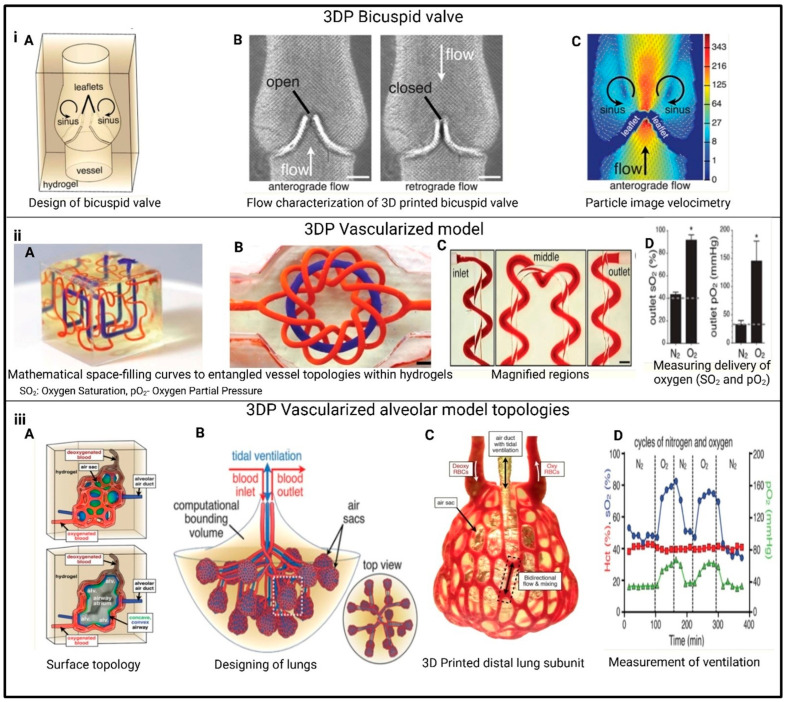
Three-dimensional design of bicuspid valve (**i-A**). Flow characterization of 3D-printed bicuspid valve [168] (**i-B**). Image representing particle size velocimetry (**i-C**). Mathematical space-filling curves to entangled vessel topologies (**ii-A,B**), showing magnified regions in (**ii-C**). Measurement of oxygen capacity (**ii-D**) (* is the oxygen delivery values). Architectural design of an alveolar model topology (**iii-A**). Elaboration of a lung-mimetic design through generative growth of the airway, offset growth of opposing inlet and outlet vascular networks, and population of branch tips with a distal lung subunit (**iii-B**). Three-dimensional printed distal lung subunit showing major parts (airduct, air sac) (**iii-C**). Measurement of ventilation (**iii-D**). Reproduced with permission from [167] under CCBY.

Grigoryan et al. [167] used PEGDA, a photopolymerizable hydrogel, as the starting material for 3DP complex geometries. PEGDA was combined with food dye additives, which served as biocompatible yet potent photo absorbers for projection stereolithography, a method that enabled the creation of intricate vascular networks and structures. The study demonstrated the successful printing of monolithic transparent hydrogels, which included efficient intravascular 3D fluid mixers and functional bicuspid valves. These advances provided intravascular and multivesicular design freedoms, allowing for the fabrication of realistic distal lung models with integrated vascular and airway spaces. The printed structures were designed to mimic the fluid dynamics of biological systems, showcasing their potential for oxygenating human red blood cells during tidal ventilation and exploring the flow and distension of proximate airways (Figure 13).

## 8. Challenges and Future Prospectus

Three-dimensional bio-printed constructs for tissue engineering often face several challenges, including balancing mechanical strength with porosity and bioactivity, optimizing scaffold geometry to enhance cellular immune interaction and nutrient transport, and ensuring long-term biocompatibility while avoiding immune responses. Traditional synthetic polymers often lack the necessary biological cues to stimulate tissue regeneration, while natural biopolymers may suffer from inadequate mechanical properties, necessitating the development of advanced composites that can integrate ceramics or synthetic reinforcements. Additionally, achieving precise control over scaffold architecture through AM parameters remains complex, as variations in filament placement and printing techniques can significantly impact mechanical performance and biological outcomes. Future prospects lie in designing biomimetic scaffolds that replicate native tissue microenvironments, incorporating bioactive molecules like growth factors or RNA therapies to direct cell differentiation, and leveraging hybrid materials that combine the biocompatibility of natural polymers with the durability of synthetics. Advances in 3D printing technologies, such as multi-material printing and dynamic scaffold systems, could further enable personalized implants with spatially graded properties, while computational modeling may optimize pore structures for vascularization and load-bearing requirements. However, clinical translation demands rigorous validation of scaffold degradation kinetics, scalability, and regulatory compliance to ensure safety and efficacy in regenerative medicine applications.

## 9. Conclusions

Additive manufacturing has revolutionized tissue engineering by enabling the fabrication of highly complex, patient-specific scaffolds with improved biomimetic properties. The advancements in AM techniques have significantly enhanced scaffold design, promoting better cellular response and tissue regeneration. While AM requires high initial investment and specialized equipment, it offers long-term cost benefits through reduced material waste, streamlined production processes, and minimized labor costs. Furthermore, the ability to manufacture scaffolds on demand reduces storage and supply chain expenses, making AM a cost-effective approach over time. However, challenges such as scalability, mechanical stability, and regulatory approval must be addressed for widespread clinical translation. Future research should focus on improving material properties, hybrid fabrication strategies, and automated post-processing to enhance the feasibility of AM in tissue engineering and regenerative medicine.

## Figures and Tables

**Table 1:** Detailed comparison of conventional methods for the preparation of scaffolds.

Method	Materials Used	Key Findings	Shortcomings	References
Solvent casting	Paracetamol, amoxicillin carboxymethyl cellulose, sodium alginate, carrageenan	Improved drug loading, enhanced mucoadhesion, improved drug retention	Potential brittleness and mechanical properties need optimization	[18]
	silver and gellan gum methacrylate film dressings	Developed nanocomposite films containing silver nanoparticles	Requires UV exposure; incomplete curing	[19]
	Phenytoin, polyvinyl alcohol, and high methoxyl pectin	Improved dissolution and rapid disintegration.	Traces of residual solvents; require careful solvent selection	[20]
	Gentamicin sulfate-loaded polycaprolactone (PCL) matrices	Enhanced drug distribution and improved anti-bacterial efficacy	Requires use of organic solvents; potential toxicity concerns	[21]
Freeze Drying	Ferulic Acid-loaded PLA and PLGA Polymeric Nanoparticles	Ocular drug delivery with enhanced antioxidant stability	Challenges in maintaining nanoparticle integrity	[22]
	Cilostazol, trehalose, maltodextrin and PEG 1500	Improving stability and bioavailability	Need to remove traces of DMSO	[23]
	Insulin- PLGA nanoparticles, Trehalose, glucose, sucrose, fructose, and sorbitol	Preserved insulin activity for 6 months	Need to optimize cryoprotectants to prevent aggregation	[24]
	Ampicillin solid lipid nanoparticles	Reduced aggregation and improved stability	Not reported	[25]
TIPS	Functionalized TIPS Microparticles	Developed functionalized TIPS microparticles for “click” conjugation, enhancing surface functionalization.	Limited exploration in surface functionalization.	[26]
	Protein-loaded PLGA TIPS microspheres	Introduced rapid formation of monodisperse porous microspheres using TIPS.	Specific to certain polymer–solvent systems only	[27]
	Rhodamine B	Rapid formation; customizable porosity	Not reported	
	Recombinant Human Growth Hormone (rhGH)	Controlled release preparation suitable for tissue engineering	Requires precise control over fabrication parameters	
Gas foaming	Poly(L-lactide-co-ε-caprolactone)/Silk Fibroin (PLCL/SF) with Strontium-EGCG MPNs (metal phenolic networks)	Simultaneous inflammation mitigation and cartilage matrix remodeling; enhanced cell infiltration	Requires precise control of MPN composition	[28]
	Poly (propylene carbonate) (PPC), starch, bioglass particles	Benign degradation byproducts; tunable porosity and mechanical properties	Potential challenges in achieving uniform pore size distribution	[11]
	Polycaprolactone (PCL)	Highly porous structure; well-interconnected pores; improved hydrophilicity and biocompatibility after plasma treatment	Requires post-treatment to enhance biocompatibility	[29]
Electrospinning	Mycophenolic Acid and collagen	Targeted drug delivery, protection from degradation, and stability in biological fluids	Complexity in fabrication leads to poor scalability	[30]
	Dexamethasone, PLA, PCL	Controlled drug release, uniform sized nanometric fiber, enhanced cell adhesion and proliferation	Potential challenges in optimizing scaffold properties for specific tissues	[31]
	Nanofibers using gelatin (core) and chitosan (shell)	Biocompatible and biodegradable scaffolds for TE and wound healing, supports cell growth and regeneration	Not reported	[32]
Electrospinning combined with Gas Foaming	Poly(L-lactide-co-ε-caprolactone)/Silk Fibroin (PLCL/SF)	Improved cell proliferation and maintenance of chondrocyte phenotype	Complex fabrication process	[33]
Electrospinning with co-axial needles	Shell (PCL/PEO) needles, forming core–shell nanofibers.	Tunable sustained release behavior, improved mechanical strength	Use of organic solvents	[34]
Sol–gel method	Titanium dioxide nanoparticles, ethanol, acetic acid, water	Enhanced photocatalytic activity due to anatase phase formation	Requires precise control of synthesis parameters	[35]
	Zinc acetate dihydrate thin films, ethanol, monoethanolamine	Uniform and transparent thin films suitable for photonic applications	Sensitivity to annealing conditions affecting film quality	[36]
	Silica sol, lipase enzyme,	Effective immobilization and preserving enzyme activity	Limitations in diffusion	[37]
Starch consolidation	Corn starch and ceramic powders	high porosity, suitable for bone TE applications	Potential brittleness due to high porosity	[38]
Starch-based polymer scaffolds via 3D printing	Cornstarch, dextran and gelatin	Scaffolds with interconnected pores for TE	Insufficient mechanical properties may be for load-bearing applications	[39]

## Data Availability

Dataset can be made available from the corresponding authors on reasonable request.

## Abbreviations

3DBST—3D bio-printed skin tissues; ALP—alkaline phosphatase; AM—additive manufacturing; Ary-Gly-Asp—arginyl-glycyl-aspartic acid; ASTM—American Society for Testing and Materials; CAD—computer-aided design; CFD—computational fluid dynamics; CFR–Code of Federal Regulations; cGMP—current Good Manufacturing Practices; CNT—carbon nanotubes; DLP—digital light processing; ECM—extracellular matrix; EXT—extrusion based technique; FDM—fusion deposition modeling; GAGs—glycosaminoglycan; GelMA—gelatin methacrylate; HUVEC—human umbilical vein endothelial cell; IJ—Inkjet; LAB—laser-assisted 3D bioprinting; MSC—mesenchymal stem cells; MTT—3-(4,5-dimethylthiazol-2-yl)-2,5-diphenyltetrazolium bromide; OCP—octacalcium phosphate; P34HB/PVA—poly-3-hydroxybutyrate-co-4-hydroxybutyrate/polyvinyl alcohol; PCL—polycaprolactone; Pus—polyurethanes; SLA—stereolithography; SLS—selective laser sintering; TE—tissue engineering.
